# SARS-CoV-2 Antibody Isotypes in Systemic Lupus Erythematosus Patients Prior to Vaccination: Associations With Disease Activity, Antinuclear Antibodies, and Immunomodulatory Drugs During the First Year of the Pandemic

**DOI:** 10.3389/fimmu.2021.724047

**Published:** 2021-08-27

**Authors:** Johanna Sjöwall, Mohammad Azharuddin, Martina Frodlund, Yuming Zhang, Laura Sandner, Charlotte Dahle, Jorma Hinkula, Christopher Sjöwall

**Affiliations:** ^1^Department of Biomedical and Clinical Sciences, Division of Inflammation and Infection/Infectious Diseases, Linköping University, Linköping, Sweden; ^2^Department of Biomedical and Clinical Sciences, Division of Molecular Medicine and Virology, Linköping University, Linköping, Sweden; ^3^Department of Biomedical and Clinical Sciences, Division of Inflammation and Infection/Rheumatology, Linköping University, Linköping, Sweden; ^4^Department of Biomedical and Clinical Sciences, Division of Inflammation and Infection/Clinical Immunology & Transfusion Medicine, Linköping University, Linköping, Sweden

**Keywords:** COVID-19, lupus (SLE), antibody response, neutralization (effect of), antinuclear antibodies, complement-immunological terms

## Abstract

**Objectives:**

Impact of the severe acute respiratory syndrome coronavirus 2 (SARS-CoV-2) pandemic on individuals with arthritis has been highlighted whereas data on other rheumatic diseases, e.g., systemic lupus erythematosus (SLE), are scarce. Similarly to SLE, severe SARS-CoV-2 infection includes risks for thromboembolism, an unbalanced type I interferon response, and complement activation. Herein, SARS-CoV-2 antibodies in longitudinal samples collected prior to vaccination were analyzed and compared with SLE progression and antinuclear antibody (ANA) levels.

**Methods:**

One hundred patients (83 women) with established SLE and a regular visit to the rheumatologist (March 2020 to January 2021) were included. All subjects donated blood and had done likewise prior to the pandemic. SARS-CoV-2 antibody isotypes (IgG, IgA, IgM) to the cell receptor-binding S1-spike outer envelope protein were detected by ELISA, and their neutralizing capacity was investigated. IgG-ANA were measured by multiplex technology.

**Results:**

During the pandemic, 4% had PCR-confirmed infection but 36% showed SARS-CoV-2 antibodies of ≥1 isotype; IgA was the most common (30%), followed by IgM (9%) and IgG (8%). The antibodies had low neutralizing capacity and were detected also in prepandemic samples. Plasma albumin (*p* = 0.04) and anti-dsDNA (*p* = 0.003) levels were lower in patients with SARS-CoV-2 antibodies. Blood group, BMI, smoking habits, complement proteins, daily glucocorticoid dose, use of hydroxychloroquine, or self-reported coronavirus disease 2019 (COVID-19) symptoms (except fever, >38.5°C) did not associate with SARS-CoV-2 antibodies.

**Conclusion:**

Our data from early 2021 indicate that a large proportion of Swedish SLE patients had serological signs of exposure to SARS-CoV-2 but apparently with a minor impact on the SLE course. Use of steroids and hydroxychloroquine showed no distinct effects, and self-reported COVID-19-related symptoms correlated poorly with all antibody isotypes.

## Introduction

The coronavirus disease 2019 (COVID-19) pandemic has caused disastrous effects worldwide and posed enormous challenges to healthcare. For patients with immune-mediated diseases on continuous treatment with immunosuppressive (or immunomodulatory) drugs, concerns have been raised regarding increased susceptibility to COVID-19 and potentially harmful effects on underlying chronic diseases (1, 2). Recently, the impact of severe COVID-19 on individuals taking disease-modifying antirheumatic drugs (DMARDs) due to inflammatory joint diseases, e.g., rheumatoid arthritis (RA), was demonstrated using Swedish register data (3). Increased risks were mainly linked to comorbidities, and the use of DMARDs (including biologics, such as cytokine-targeted therapies) did not greatly influence the risk of severe COVID-19 infection or death. However, data on other rheumatic conditions, e.g., systemic lupus erythematosus (SLE), are still scarce.

SLE represents a prototype disease of systemic autoimmunity in which immune complexes or cytotoxic antibodies may give rise to tissue damage and organ failure (4). Clinical features and laboratory abnormalities typical of active SLE show several similarities with COVID-19. A dysregulated type I interferon (IFN) system is typical of SLE (5–7). Type I IFNs are key components of the innate and adaptive immune responses to new pathogens, and their pivotal role in antiviral immunity is well established, including unbalanced inflammatory responses to severe acute respiratory syndrome coronavirus 2 (SARS-CoV-2) (8, 9). Preliminary data suggest that patients with SLE do not have an increased risk of SARS-CoV-2 infection, or severe COVID-19, compared with the general population (2, 10, 11). Still, it cannot be excluded that COVID-19 leads to an increased rate of SLE flares, which has been shown to be the case with other infections or challenges to the immune system (12–14). Furthermore, COVID-19 has been associated with activation of the complement system as well as the development of autoantibodies in hospitalized patients; manifest autoimmune disease related to these newfound autoantibodies and complement consumption has also been observed (15–18). Another feature of COVID-19, resembling SLE and antiphospholipid syndrome (APS), is the increased risk of thromboembolic events (15, 19). Thereto, early in the pandemic, the use of the cornerstone drug for SLE, hydroxychloroquine (HCQ), was suggested to have antiviral effects, but current data do not support its use in COVID-19. The impact on the risk of COVID-19 regarding other drugs used in SLE, e.g., B-cell-targeted therapies, is yet unclear. In multiple sclerosis (MS), the use of rituximab (anti-CD20) is associated with a two to threefold higher risk for severe COVID-19, and the risk increases with the duration of rituximab therapy (20).

To gain an increased understanding of the immune response towards SARS-CoV-2 in patients with SLE, we focused on the following aims: (I) to assess to which extent well-characterized cases with established SLE have been exposed to, and managed to mount an IgG, IgA, or IgM antibody response to, SARS-CoV-2 during the first year of the pandemic (prior to vaccination); (II) to investigate the neutralizing capacity of the detected SARS-CoV-2 antibodies; and to evaluate if the serological signs of COVID-19 were related to (III) progression of SLE or (IV) antinuclear antibody (ANA) levels and (V) the use of immunomodulatory drugs. To address these questions, we took advantage of a previously described cohort of Swedish SLE patients with longitudinal follow-up (21).

## Materials and Methods

### Subjects

The study population consisted of 100 patients (83 females, 17 males) with established SLE who had a regular physical visit to the Rheumatology unit at Linköping University Hospital, Sweden, from March 2020 to January 2021. All patients fulfilled the 1982 American College of Rheumatology (ACR) and/or the 2012 Systemic Lupus International Collaborating Clinics (SLICC) classification criteria for SLE and had previously been included in the prospective follow-up program KLURING (a Swedish acronym for *Clinical LUpus Register In North-eastern Gothia*) at the Department of Rheumatology, Linköping University Hospital, as described in detail (21). SLE disease activity was assessed using SLE disease activity index-2000 (SLEDAI-2K) and physician’s global assessment (PGA) (22). Irreversible organ damage, required to have been persistent for ≥6 months, was recorded annually by SLICC/ACR damage index (SDI), which encompasses damage in 12 defined organ systems (23). Health-related quality of life (HRQoL) was obtained using the EuroQoL-5 dimensions (EQ-5D) (24). The participating patients donated blood consecutively at their regular visit during the pandemic and had done likewise at another visit to the Rheumatology unit prior to the pandemic. Thus, a corresponding prepandemic serum sample (from August 2015 to November 2019) was available from each participating subject. All serum samples were stored at −70°C until analysis. Ongoing pharmacotherapy, including daily prednisolone dose was registered at each visit. Detailed characteristics of the study population are shown in **Table 1**.

**Table 1:** Characteristics of the 100 included patients with SLE.

	Prepandemic (n = 100)	Pandemic (n = 100)
*Background variables*		
Females (*n*)	83	83
Age at blood sampling [*mean years* (range years)]	48.7 (19–87)	51.3 (20–90)
SLE duration at sampling [*mean years* (range years)]	12.5 (0–42)	15.1 (1–47)
Caucasian ethnicity (*n*)	86	86
Ever smoker (former or current) (*n*)	42	49
Body mass index [*mean kg/m^2^* (range)]	26.6 (17.0–42.7)	27.1 (17.1–45.7)
*Disease variables*		
SLEDAI-2K [*mean score* (range)]	2.4 (0–16)	1.9 (0–24)
SLICC/ACR damage index (SDI) [*mean score* (range)]	1.1 (0–6)	1.3 (0–8)
Physician’s global assessment [*mean score* (range)]	0.4 (0–3)	0.3 (0–2)
EQ–5D (*mean score* (range))	0.66 (−0.24–1)	0.69 (−0.48–1)
Erythrocyte sedimentation rate [*mean mm/h* (range)]	20.0 (2–108)	18.3 (1–106)
C-reactive protein [*mean mg/L* (range)]	6.8 (2.5–172)	6.1 (2.5–166)
Complement protein 3 [*mean g/L* (range)]	1.1 (0.4–1.8)	0.98 (0.5–1.9)
Complement protein 4 [*mean g/L* (range)]	0.19 (0.02–0.55)	0.18 (0.02–0.53)
Albumin [*mean g/L* (range)]	NE	39.1 (28–50)
Anti-dsDNA antibody levels [*mean IU/ml* (range)]	95.6 (2–900)	101.3 (0–1081)
Blood group 0* (*n*)	38	38
Blood group A (*n*)	37	37
Blood group B (*n*)	11	11
Blood group AB (*n*)	4	4
Rh+ (*n*)	78	78
Rh− (*n*)	12	12
*Pharmacotherapy*		
Hydroxychloroquine (*n*)	77	73
Azathioprine (*n*)	8	8
Mycophenolate mofetil (*n*)	21	23
Rituximab (*n*)	2	3
Cyclophosphamide (*n*)	1	0
Sirolimus (*n*)	4	3
Belimumab (*n*)	0	4
Daily prednisolone dose (*mean mg*)	4.5 (0–30)	3.5 (0–15)
*1982 American College of Rheumatology classification criteria (ACR-82)*		
Cases meeting ≥4 ACR-82 criteria (*n*)	80^#^	82^#^
Number of fulfilled ACR-82 criteria [*mean* (range)]	4.7 (3–9)	4.8 (3–9)
1. Malar rash (*n*)	33	35
2. Discoid rash (*n*)	11	12
3. Photosensitivity (*n*)	48	48
4. Oral ulcers (*n*)	14	14
5. Arthritis (*n*)	78	80
6. Serositis (*n*)	29	29
7. Renal disorder (*n*)	34	34
8. Neurologic disorder (*n*)	10	10
9. Hematologic disorder (*n*)	58	62
10. Immunological disorder (*n*)	57	61
11. Antinuclear antibody^†^ (*n*)	99	99

NE, not estimated.

*Blood group data available for 90 participants.

^#^All patients that did not fulfil ACR-82 met the 2012 SLICC classification criteria.

^†^Positive by indirect immunofluorescence microscopy on HEp-2 cells.

### Routine Laboratory Measurements and Autoantibody Analyses

Blood cell counts, plasma creatinine, creatine kinase, complement protein 3 (C3), C4, C-reactive protein (CRP), erythrocyte sedimentation rate (ESR), and urinalysis were measured as part of a clinical routine both at the prepandemic and the pandemic visits. In addition, IgG-ANA fine specificities, including antidouble-stranded DNA (dsDNA) and 13 additional autoantibodies, were analyzed by FIDIS™ Connective Profile, Solinium software version 1.7.1.0 (Theradiag, Croissy-Beaubourg, France) in February 2021 at the Clinical Immunology Laboratory, Linköping University Hospital in collected sera from both visits (25). This addressable laser bead assay (ALBIA) measures autoantibodies to Ro52/SSA, Ro60/SSA, La/SSB, Smith antigen (Sm), Smith/ribonucleoprotein (Sm/RNP), U1RNP, scleroderma 70 kD antigen (Scl-70), dsDNA, histone, ribosomal P protein (RibP), centromere protein B (CENP-B), polymyositis/systemic sclerosis complex (PmScl), histidyl-tRNA synthetase (Jo-1), and proliferating cell nuclear antigen (PCNA). According to the manufacturer’s instructions, a cutoff for each antibody specificity at 40 IU/ml was applied. Sera collected prior to and during the pandemic were analyzed in parallel to avoid interassay variation.

### Self-Reported Symptoms Associated With COVID-19

Using a questionnaire, patients were interviewed by telephone regarding COVID-19-associated symptoms during the study period: fever >38.5°C, headache, hypogeusia, hyposmia, cough, dyspnea, sore throat, rhinorrhea, myalgia, fatigue, diarrhea, nausea, vomiting, abdominal pain, deep vein thrombosis, and pulmonary embolism.

### Review of Medical Records

Digital medical records were reviewed with respect to confirmed COVID-19 by detection of SARS-CoV-2 RNA in the respiratory tract, hospitalization, and severity of illness category according to the National Institute of Health, and blood group according to the Rhesus (Rh) and the AB0 group system, respectively.

### SARS-CoV-2 PCR Assay

The RealTime SARS-CoV-2 assay using nasopharyngeal swab specimens was performed at the Clinical Microbiology Laboratory, Linköping University Hospital, according to the Emergency Use Authorization product insert (26). Tests were considered negative if no genome had been detected over 44 cycles.

### SARS-CoV-2 Antibody Assay

Enzyme-linked immunosorbent assays (ELISA) were mainly performed as described elsewhere (27, 28), with a slight modification as presented below. SARS-CoV-2 S1-spike protein (Wuhan strain, 2019) was used as soluble antigen on 96-well microplates (0.5 µg/ml) in PBS (Sino Biological, Eschborn, Germany). The antigen coated plates were blocked for 60 min at 37°C with 5% fat-free milk buffer. Sera from the SLE cases and patients with previously confirmed COVID-19 infection, as well as from positive and negative controls, were diluted in PBS-Tween 20 (0.05%) with 2.5% fat-free milk buffer. Serum dilutions were added to the coated plate wells and incubated 90 min at 37°C. Conjugates against antihuman IgG-HRP (BioRad, Richmond, CA, USA), antihuman IgM (Abcam, NordicBiosite, Täby, Sweden), or antihuman IgA-HRP (Nordic BioSite, Täby, Sweden) were added to separate wells with diluted serum samples and incubated 90 min at 37°C. Finally, 0.003% H_2_O_2_/o-phenylene diamine substrate (Sigma-Aldrich, St. Louis, MA, USA; 0.4 mg/ml) was added, and the plates were kept in darkness at room temperature for 30 min before 2.5 M H_2_SO_4_ was used as stop solution. Plates were read at an optical density (OD) of 490 nm. A standard curve was used to determine arbitrary units (AU), estimating quantitative levels of SARS-CoV-2-specific antibodies. Cutoff for positive tests was defined as an OD above the 3rd standard deviation of samples from healthy donors collected before the pandemic. All SLE samples were analyzed in parallel and blinded regarding whether they had been collected before or during the pandemic.

### Inhibition Assays

Serum samples with strong IgA and/or IgM SARS-CoV-2 reactivity as judged by our assay were used for blocking experiments. The sera were preincubated 1 h at 37°C with increasing concentrations (0.1, 1, and 10 µg/L) of SARS-CoV-2 S1-spike protein or with irrelevant antigens: influenza A or bovine serum albumin (BSA). The samples were thereafter treated and analyzed as described above.

### Commercial IgA SARS-CoV-2 Antibody ELISA

To cross-validate the in-house ELISA, we tested the prepandemic and pandemic samples with an FDA-approved *in vitro* diagnostic ELISA kit (IgA anti-SARS-CoV-2 ELISA, EI-2606-9601A, EUROIMMUN AG, Kriens, Switzerland) which provides a semiquantitative *in vitro* determination of human antibodies of the immunoglobulin class IgA against SARS-CoV-2 in serum, EDTA, heparin, or citrate plasma (29). The assay was performed according to the manufacturer’s instruction. Briefly, all the reagents were brought to room temperature approximately 30 minutes before use. One hundred microliters of the diluted samples (prepandemic and pandemic) was transferred to the individual microplate wells and incubated for 60 min at 37°C. Immediately after sample incubation, the microplate was washed with washing buffer three times, leaving the wash buffer in each well for 30 to 60 s per washing cycle, followed by 100 µl incubation of enzyme conjugate for 30 min at 37°C. Another round of washing as described earlier was carried out. One hundred microliters of substrate solution was added and incubated for another 30 min this time at room temperature and away from direct sunlight. The reaction was stopped by adding 100 µl of stop solution into each well and finally photometric measurement of the color intensity was recorded at 450 nm wavelength and a reference wavelength at 650 nm. The extinction of the calibrator (provided in the kit) defines the upper limit of the reference range of noninfected persons (cutoff) recommended by the manufacturer.

Semiquantitative results were calculated using a ratio of the extinction of the control or patient sample over the extinction of the calibrator. The ratio was estimated according to the following formula:

Ratio=Extinction of the control or patient sample/Extinction of calibrator

The manufacturer recommended the following interpretation of the results:

Ratio<0.8=Negative

Ratio≥0.8−<1.1=Borderline

Ratio≥1.1=Positive

### Microneutralization Assay

Serum neutralization assay was performed as described elsewhere (30). Briefly, heat-inactivated serum including positive and negative controls was serially diluted in twofold steps from 1:4, 1:8, 1:16 until 1:1,024 in MEM-2% HI fetal calf serum (FCS). Serum dilutions was added 75 µl/well in duplicate wells (96-well flat well cell culture plate). Virus (SARS-CoV-2, 2020-nCoV [SARS-CoV-2-Iso_LiU-Human-2020-03-04-Swe]) was added 75 µl/well at a concentration of 100–130 PFU/ml and incubated for 1 h at 37°C 5% CO_2_. After incubation, the serum/virus mixture was added onto wells with 5 × 10^4^ Vero E6 cells/well in 100 µl MEM with 2% HI FSC. The plates were kept at 37°C 5% CO_2_ for 96 h before examined in the microscope for ratio of healthy cells vs virus-induced cell cytotoxic effect (CPE) areas. Cells were fixed for 30 min and stained with 0.1% crystal violet. Serum dilutions that showed 50% inhibition of CPE was given as the neutralization titer.

### Inhibition of S1-Spike Protein-Binding to ACE2 Cell Receptor *In Vitro*

Analysis was performed as previously described (31). In brief, 96-well microplates were coated with 1 µg/ml recombinant human ACE2-protein (in PBS pH 7.4) over night at 4°C. Heat-inactivated serum samples diluted 1:50, 1:150, and 1:450 were mixed with 1 µg/ml soluble recombinant S1-spike protein in duplicate microwells in 100 µl/well and incubated at 37°C for 30 min before being transferred to the ACE2-coated microwells. Plates were incubated for 1 h at 37°C. Plates were washed with PBS-Tween 20 (0.05%). HRP-labeled antihuman S1-spike monoclonal antibody was added (100 µl/well) and incubated for 1 h at 37°C. Plates were washed and substrate o-phenylene diamine/0.003% H_2_O_2_ was added as substrate and incubated at RT for 20 min. Substrate reaction was stopped with 100 µl/well 2.5 M H_2_SO_4_ before plates were read at optical density (OD) 490 nm. Controls with no serum and human serum lacking S1-spike binding was used as negative controls, and human serum with high SARS-CoV-2 neutralization titer (1,024) was used as positive control. Serum samples with 50% of more inhibition of ACE2-S1-spike protein-binding OD 490 nm reactivity was considered a significantly inhibiting serum titer.

### Avidity Assay

Avidity analysis of serum immunoglobulin binding to viral antigens by ELISA was performed as previously reported (32).

### Statistics

For comparisons of biomarker levels between groups, the Mann-Whitney *U*-test was used. Associations between seropositivity (categorical variable) and self-reported symptoms, SLE phenotypes (ACR criteria), and disease activity were examined with the *χ*^2^ test, or Fisher’s exact test when appropriate (*n* ≤ 5). When antibody levels were compared before and during the pandemic, Wilcoxon-matched paired signed rank test was used. Spearman’s correlation was employed for all correlation analyses. *p*-Values ≤0.05 were considered statistically significant. Statistical analyses were performed using the SPSS software ver. 26.0.0.0 (SPSS Inc., Chicago, IL, USA) or GraphPad Prism ver. 8.4.3 (GraphPad Software Inc., San Diego, CA). Graphs were created using GraphPad Prism ver. 8.4.3 (GraphPad Software).

### Ethical Considerations

This study was carried out in accordance with the Declaration of Helsinki. Written informed consent was obtained from all participants. The study protocol was approved by the regional ethics review board in Linköping (Decision number M75-08/2008).

## Results

### SARS-CoV-2 IgG, IgA, and IgM Antibodies Pre- and During the Pandemic

In total, four patients (4%) had confirmed COVID-19 during the study period and one of them was hospitalized in the intensive care unit for 42 days and needed mechanical ventilation for 29 days. The cycle threshold (Ct) values of the four positive samples were 16.79, 31.83, 31.94, and 35.92. During the study period, 26 patients tested negative at least once.

**Figure 1:**
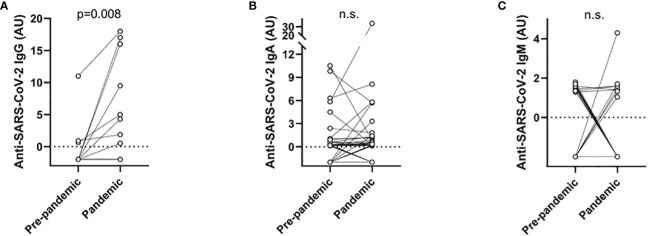
Arbitrary units (AU) of SARS-CoV-2 antibodies in the sera of the 100 included patients with SLE demonstrated for each isotype: IgG **(A)**, IgA **(B)**, and IgM **(C)** before and during the pandemic. Only SARS-CoV-2 IgG levels were significantly higher during the pandemic compared with prepandemic samples. n.s., not significant.

Specific blood group, according to the AB0 and Rh systems, did not associate with either confirmed COVID-19 or presence of SARS-CoV-2 antibodies. A history of SARS-CoV-2 RNA positivity during the pandemic did not associate with presence of any of the SARS-CoV-2 antibody isotypes. During the pandemic, 36% had detectable SARS-CoV-2 antibodies of ≥1 isotype; IgA was the most common (30%), followed by IgM (9%) and IgG (8%). No significant gender difference was detected in SARS-CoV-2 antibodies. However, trends were found with higher percentage of men being positive for both IgG (*p* = 0.10) and ≥1 isotype (*p* = 0.11). SLE duration and age at sampling were not associated with SARS-CoV-2 antibody status. As illustrated in **Figures 1A–C**, SARS-CoV-2 IgG was significantly higher during than prior to the pandemic, whereas the IgA and IgM isotypes did not differ significantly. Several patients showed detectable SARS-CoV-2 antibody levels during the prepandemic period.

Self-reported respiratory symptoms (cough, dyspnea, sore throat, rhinorrhea) did not associate with presence of any SARS-CoV-2 antibody isotype. However, self-reported fever (>38.5°C) associated significantly with the presence of SARS-CoV-2 IgG (*p* = 0.03) during the pandemic but not with IgA or IgM. None of the other self-reported symptoms (headache, hypogeusia, hyposmia, myalgia, fatigue, diarrhea, nausea, vomiting, abdominal pain, deep vein thrombosis, and pulmonary embolism) associated with antibody findings.

### Dose-Dependent Reduction of IgA/IgM Reactivity by Preincubation With S1-Spike Protein

Inhibition of SARS-CoV-2 IgA and IgM reactivity by preincubation with S1-spike protein or irrelevant proteins was evaluated in a selected collection of serum samples yielding strong reactivity in the assays described above. As illustrated in paired samples from three patients in **Supplementary Figure S1A–F**, a dose-dependent reduction in antibody binding was achieved for both IgA and IgM following preincubation with S1-spike protein. For IgA, inhibition of >50% was observed in 11 of 13 (85%) samples and >80% in 8/13 (62%). For IgM, inhibition of >50% was demonstrated in nine of 10 samples (90%) and >80% in seven of 10 (70%).

### Agreement Between the In-House IgA Assay and EUROIMMUN SARS-CoV-2 IgA ELISA

The level of agreement between the in-house IgA assay and the commercial SARS-CoV-2 IgA ELISA was evaluated in 50 paired samples originating from 25 patients of the study population. As demonstrated in **Table 2**, the differences between prepandemic and pandemic samples were strongly correlated (*p* = 0.0001). The correlation between the assays in prepandemic samples was also highly significant (*p* = 0.005) whereas it did not reach statistical significance in the pandemic samples. The concordance (defined in **Table 2**) was fair, 76% for all samples. The entire data set of the commercial SARS-CoV-2 IgA ELISA is shown in **Supplementary Figure S2A–G**.

**Table 2:** Correlation analyses and concordance between results of the IgA in-house S1 spike assay and the EUROIMMUN IgA ELISA.

	Number of samples	Percentage positive by assay	Concordance^*^	Spearman’s rho	*p*-Value
**Prepandemic to pandemic: change between paired samples** (in-house, AU, *vs.* EUROIMMUN, ratio)	25x2	N/A	N/A	0.78	0.0001
**Prepandemic samples compared** (in-house, AU, *vs.* EUROIMMUN, ratio)	25		72%	0.54	0.005
*In-house*		19/25 (76%)			
*EUROIMMUN*		12/25 (48%)			
**Pandemic serum samples compared** (in-house, AU, *vs.* EUROIMMUN, ratio)	25		80%	0.36	0.07
*In-house*		22/25 (88%)			
*EUROIMMUN*		15/25 (60%)			
**All samples compared** (in-house, AU, *vs.* EUROIMMUN, ratio)	50		76%	0.47	0.0005
*In-house*		41/50 (82%)			
*EUROIMMUN*		27/50 (54%)			

AU, arbitrary units; N/A, not applicable.

*The sum of double-positive samples and double-negative samples, divided by the total number of samples, multiplied by 100.

### Neutralizing Capacity of Detected SARS-CoV-2 Antibody Isotypes

In a selected serum sample collection (20 serum samples, originating from 13 patients), where evident ELISA-seropositive immunoglobulin reactivity against the recombinant S1-spike protein was observed, the virus-neutralizing activity *in vitro* was investigated. All tested serum samples had no, or very low neutralizing reactivity; below eight in neutralizing serum titer *in vitro* with the used virus (**Table 3**). The positive control showed neutralizing titer of 1,024 and negative controls were negative (<8). The same sera were subsequently tested in a S1-spike- and recombinant ACE2 receptor-binding inhibition assay *in vitro*. The inhibiting capacity was low, shown to be between 5% and 42% (median 10%, range 0%–42%). Serum from SARS-CoV-2 seropositive controls showed >90% inhibition (91%–96%) and negative control sera <15% inhibition (median 6%, range 0%–14%). Serum immunoglobulin avidity was tested against the recombinant S1 protein with ELISA-positive IgG, IgA, and IgM serum samples, and the avidity index (AI) of reactivity among the patient sera were strongest with the IgG isotype (median 0.63, range 0.5–0.83); avidity index of the IgA and IgM isotypes were considerably lower (for IgA, median was 0.23, range 0.19–0.26; for IgM, median 0.21, range 0.18–0.27). The positive control serum showed median IgG avidity index of 0.99, and for IgA, a median avidity of 0.81 was observed.

**Table 3:** Serological virus-inhibition analysis of S1 ELISA-reactive sera.

Serum samples	Number of samples	NT (>8)	S1-ACE2 block (>25%)	AI (median)		
				IgG	IgA	IgM
SARS-CoV-2 positive: SLE	20 (13 patients)	0/20	1/20	0.63	0.22	0.21
Negative control	8	0/8	0/8	<0.1	<0.1	<0.1
Positive control	4	4/4	4/4	0.99	0.84	N/A

NT, neutralization assay; AI, avidity index; N/A, not applicable.

### SARS-CoV-2 Antibodies in Relation to SLE Phenotypes, HRQoL, and Disease Activity

Regarding SLE phenotypes, photosensitivity (ACR criterion 3) associated inversely with presence of SARS-CoV-2 IgA (*p* = 0.05). Immunological disorder (ACR criterion 10) associated inversely with both presence of SARS-CoV-2 IgM (*p* = 0.01) and with any SARS-CoV-2 antibody isotype (*p* = 0.03). Limited accrual of organ damage was observed (see SDI, **Table 1**); individuals who acquired damage were not SARS-CoV-2 antibody positive to a higher extent than those with unchanged SDI.

**Figure 2:**
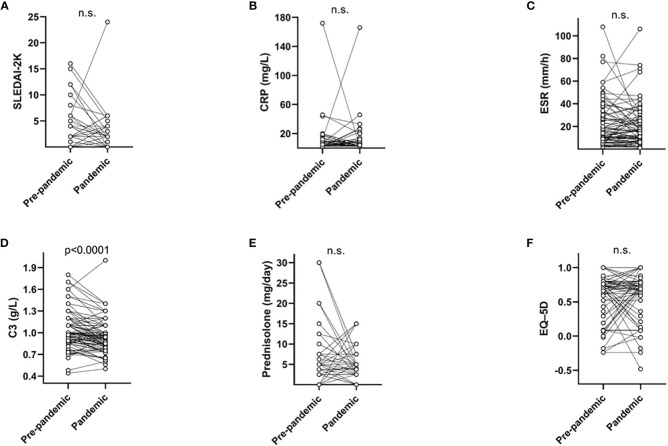
Global SLE disease activity (SLEDAI-2K) **(A)**, C-reactive protein (CRP) **(B)**, erythrocyte sedimentation rate (ESR) **(C)**, Complement protein 3 (C3) **(D)**, daily prednisolone dose **(E)**, and EuroQoL-5 dimensions (EQ–5D) **(F)** shown for the 100 included patients with SLE before and during the pandemic. Only the C3 levels were significantly lower during the pandemic compared with prepandemic samples. n.s., not significant.

Global SLE disease activity assessed at the two visits did not differ significantly, either by assessment of SLEDAI-2K (**Figure 2A**) or PGA. Systemic inflammation detected by CRP and ESR were also similar (**Figures 2B, C**). However, signs of increased complement consumption were observed with significantly decreased C3 levels (**Figure 2D**) whereas the reduction of C4 did not meet statistical significance. The daily use of prednisolone did not differ and neither did the HRQoL assessed by the EQ–5D (**Figures 2E, F**). Plasma albumin (*p* = 0.04) and anti-dsDNA (*p* = 0.003) levels during the pandemic were lower in patients with positive SARS-CoV-2 antibodies (of at least one isotype) compared with negative cases.

### ANA Levels Pre- and During the Pandemic *vs.* SARS-CoV-2 Antibodies

**Figure 3:**
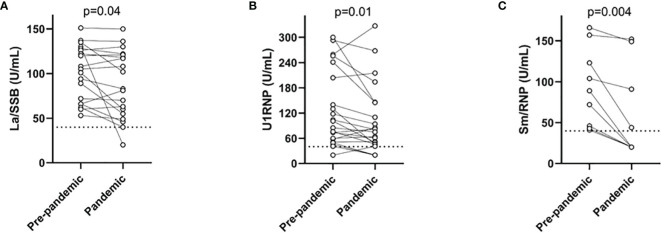
Levels of three antinuclear antibodies (ANA) targeting extrachromosomal antigens demonstrated before and during the pandemic. Significantly lower levels of La/SSB **(A)**, U1RNP **(B)**, and Sm/RNP **(C)** were found during the pandemic compared with prepandemic samples. To increase the readability, only patients with at least one sample above the cutoff of the assay (before or during the pandemic) are shown.

None of the samples contained antibodies to Jo-1 or Scl-70. Antibodies to Ro52/SSA, Ro60/SSA, Sm, dsDNA, histone, RibP, CENP-B, PmScl, and PCNA were not statistically different when prepandemic and pandemic samples of antibody-positive individuals were compared. However, ANA targeting three extrachromosomal antigens decreased significantly during the pandemic (**Figures 3A–C**). As demonstrated, lower values were achieved for most samples regarding La/SSB where 13/19 (68.4%) samples decreased, for U1RNP 15/22 (68.2%) samples decreased, and for Sm/RNP 9/9 (100%) samples decreased. However, when also taking the intra-assay variation of the method into account: 6/19 (31.6%) La/SSB-positive patients decreased and none increased; 8/22 (36.4%) U1RNP-positive patients decreased and 3/22 (13.6%) increased; and 4/9 (44.4%) Sm/RNP-positive patients decreased and none increased.

**Table 4** illustrates the associations between each ANA specificity and presence of SARS-CoV-2 antibody isotypes before and during the pandemic. Presence of anti-Sm was significantly associated with SARS-CoV-2 IgG in both prepandemic (*p* = 0.008) and pandemic (*p* = 0.03) samples. Presence of anti-dsDNA was inversely associated with SARS-CoV-2 IgA only during the pandemic (*p* = 0.02).

**Table 4:** Associations between ANA specificities and SARS-CoV-2 antibody isotypes in SLE patients before and during the pandemic.

		*SLE: prepandemic (n = 100)*			*SLE: pandemic (n = 100)*		
SARS-CoV-2 isotypes		IgG (*n* = 4)	IgA (*n* = 31)	IgM (*n* = 13)	IgG (*n* = 8)	IgA (*n* = 30)	IgM (*n* = 9)
Ro52/SSA	+	1/33	5/33	5/33	1/29	6/29	1/29
	−	3/67	26/67	8/67	7/71	24/71	8/71
Ro60/SSA	+	1/35	7/35	4/35	1/30	8/30	2/30
	−	3/65	24/65	9/65	7/70	22/70	7/70
La/SSB	+	0/19	5/19	4/19	0/17	5/17	3/17
	−	4/81	26/81	9/81	8/83	25/83	6/83
Sm	+	1/3	0/3	0/3	1/2	1/2	0/2
	−	3/97	31/97	13/97	7/98	29/98	9/98
Sm/RNP	+	0/9	3/9	1/9	0/4	2/4	0/4
	−	4/91	28/91	12/91	8/96	28/96	9/96
U1RNP	+	1/22	6/22	2/22	2/19	5/19	1/19
	−	3/78	25/78	11/78	6/81	25/81	8/81
dsDNA	+	0/41	16/41	5/41	0/37	6/37	0/37
	−	4/59	15/59	8/59	8/63	24/63	9/63
RibP	+	0/3	0/3	0/3	1/5	2/5	0/5
	−	4/97	31/97	13/97	7/95	28/95	9/95
CENP-B	+	0/3	0/3	0/3	0/4	1/4	1/4
	−	4/97	31/97	13/97	8/96	29/96	8/96
PmScl	+	0/4	2/4	1/4	0/5	2/5	1/5
	−	4/96	29/96	12/96	8/95	28/95	8/95
PCNA	+	0/5	2/5	2/5	0/3	1/3	0/3
	−	4/95	29/95	11/95	8/97	29/97	9/97
Histone	+	0/9	3/9	1/9	0/10	0/10	0/10
	−	4/91	28/91	12/91	8/90	30/90	9/90
*p* = 0.008							
*p* = 0.02							
*p* = 0.03							
Division by zero							

### SARS-CoV-2 Antibodies in Relation to Immunomodulatory Drugs

During the pandemic, potential associations between SARS-CoV-2 antibodies and ongoing immunomodulatory drugs were investigated. Presence of SARS-CoV-2 IgA was associated with the use of mycophenolate mofetil (*p* = 0.04), but none of the other DMARDs were significantly associated with the presence of SARS-CoV-2 antibodies.

## Discussion

Serological testing is a cornerstone in our understanding of infections and immune responses. A reliable immunoassay should be both sensitive and specific and perform well not only among healthy individuals but also in patient groups with immune-mediated diseases (33). Subsequently, the need of reliable SARS-CoV-2 antibody assays is extensive and crucial for opening societies after lockdowns and permit traveling. Furthermore, as the vaccination programs against COVID-19 progress worldwide, the requirement of credible SARS-CoV-2 antibody assays (to demonstrate a successful vaccine response) will probably remain over the next couple of years.

The present study was primarily undertaken to extend our knowledge of the adaptive immune response against SARS-CoV-2 in patients with established SLE, of which the great majority (92%) were taking immunosuppressive or immunomodulatory drugs. Such treatments, e.g., glucocorticoids (used by 66% during the pandemic) or mycophenolate mofetil (23%), could potentially result in decreased ability to recover from the infection, establish immunity or respond to vaccinations (34). In contrast though, a recent paper from the USA showed that most patients with SLE and confirmed COVID-19 were able to produce and maintain an IgG response despite the use of a variety of DMARDs, providing reassurance about the efficacy and durability of humoral immunity and possible protection against reinfection with SARS-CoV-2 (35). However, Saxena et al. did not have prepandemic samples available for comparison and did not report potential associations with ANA (35). However, studying the humoral immune response to SARS-CoV-2 in SLE is not only of relevance due to potential effects of pharmacotherapies, the pathogenesis of SLE is characterized by B-cell hyperactivity and reflected by the large amount of autoantibodies described (36). In addition, similarities between severe COVID-19 and SLE, such as risks for thromboembolism, an unbalanced type I IFN response and complement activation have been observed, resulting in implementation of regular use of anticoagulation and glucocorticoids in the clinical management of patients with COVID-19 (6, 7). A major strength of this study was that the SLE patients were their own controls as prepandemic samples were available and analyzed in parallel with samples collected during the pandemic.

Our data from early 2021, obtained prior to the introduction of vaccines, indicate that a large proportion of Swedish patients with SLE had serological signs of exposure to SARS-CoV-2, seemingly with poor correlation to COVID-19-related symptoms. To validate the reliability of our in-house assay, a commercially available and FDA-approved diagnostic IgA SARS-CoV-2 antibody kit was used. The assays showed a reassuring concordance in both prepandemic and pandemic samples (72%–80%), and the inhibition tests yielded dose-dependent reduction of IgA/IgM antibody reactivity following preincubation with S1-spike protein. Still, almost all analyzed antibody-positive samples showed a low neutralizing capacity indicating low-affinity antibodies with uncertain protective effect against SARS-CoV-2. Interestingly, SARS-CoV-2 antibodies (particularly IgA and IgM) were also detected in corresponding samples collected ahead of the pandemic. Whether this represents an entirely unspecific “sticky” immunoglobulin response, exposure to previous corona viruses or signs of interference with autoantibodies in the immunoassays remains an open question. Since SLE is a condition characterized by a broad repertoire of circulating autoantibodies, it is not unlikely that this group of patients in general may be more prone to produce antibodies targeting various antigens, including coronaviruses, even in the absence of COVID-19. In line with this, it was recently demonstrated that certain infections (e.g., malaria, schistosomiasis, and dengue) may yield unreliable results in rapid diagnostic COVID-19 antibody tests (37). Some betacoronaviruses have been described as capable of inducing ELISA and Western blot cross-reactive anti-SARS-CoV-2 serum responses, but in general not cross-neutralizing antibodies (38–40). The coronavirus 229E appears to be an exception (41, 42). In our study, the SARS-CoV-2 IgG isotype was less often found in prepandemic samples and was in addition the only antibody that significantly associated with self-reported symptoms (body temperature >38.5°C) during the pandemic. A meta-analysis recently concluded that blood group A may be associated with a higher risk of severe SARS-CoV-2 infection compared with group 0, which usually associates with a lower risk (43). We could not demonstrate any clear associations of specific blood group (according to the AB0 and Rh systems) with PCR-confirmed COVID-19, or with presence of SARS-CoV-2 antibodies, but larger study populations are probably needed to confirm such associations. Neither did we find any clear differences regarding SARS-CoV-2 antibody positivity related to sex, age, or SLE duration. In contrast, an age-related serum half-life of anti-SARS-CoV-2 IgM and IgG has previously been reported in the general population and severity of the infection may also be of relevance (44, 45). An obvious limitation of the study is that the proportion of subjects infected by SARS-CoV-2 is unknown. In Sweden, PCR testing of individuals with symptoms of infection was not introduced in routine for the general population until June of 2020 (46). Thus, the four patients (4%) with PCR-confirmed COVID-19 during the study period indisputably represent an underestimation, especially since the infection may well pass without symptoms.

Presence of rheumatoid factor often challenges the specificity of immunoassays and is, in general, a common suspect of false positive tests. This was recently highlighted also for different immunoassays of serological SARS-CoV-2 testing in patients with chronic inflammatory diseases, whereof most of the investigated samples originated from patients with MS and RA (47). A subgroup of rituximab-treated SLE patients was included, but rheumatoid factor did not seem to have a major impact on the SARS-CoV-2 test results in SLE. Herein, we further extended the knowledge by analyzing ANA and their potential interference with SARS-CoV-2 (shown in **Table 4**). Solely, the presence of anti-Sm showed a significant association with SARS-CoV-2 IgG in prepandemic as well as pandemic samples. Furthermore, anti-dsDNA was inversely associated with SARS-CoV-2 IgA during the pandemic and samples positive for any SARS-CoV-2 antibody isotype contained significantly lower levels of anti-dsDNA. Thus, possibly except for anti-Sm, our data indicate that the presence of specific SLE autoantibodies does not interfere with detection of SARS-CoV-2 IgG.

Regardless of SARS-CoV-2 antibody status, we observed a significant decrease during the pandemic of three specific autoantibodies targeting extrachromosomal antigens (La/SSB, U1RNP, and Sm/RNP). These reductions remained significant also when the variation of the method was considered and did not coincide with additional use of immunosuppression in the patients with declining antibody levels. Overall, clinical disease activity assessed by SLEDAI-2K and PGA remained stable. Neither did the systemic inflammation (ESR or CRP) differ over time. However, C3 decreased significantly during the pandemic and a similar trend was observed for C4. The latter usually represents increased activation of the complement pathway following immune complex deposition (48, 49). If accompanied by positive anti-dsDNA in patients with SLE, this observation is referred to as “serologically active clinically quiescent” (50). The findings of diminishing ANA levels in pandemic, compared with prepandemic, samples were unexpected. Potential reasons could be increased isolation during the pandemic and meticulous adherence to advice of social distancing that overall might have led to less infections and challenges to the immune system (13, 14).

Some limitations deserve to be mentioned. The size of the study population was limited, especially as only 4% tested positive with PCR. Due to the lack of general testing in Sweden during the beginning of the pandemic, the percentage of truly infected subjects remains unknown. Thus, associations between “confirmed” infection, laboratory variables and clinical parameters should be interpreted with caution. Furthermore, we only evaluated the humoral and not the cell-mediated immune response to SARS-CoV-2. Ethnicity of the study population constitutes another limitation. Mainly Caucasian patients were enrolled, and as ethnicity is well known to affect SLE severity, extrapolation of our results to other populations should be done with caution (51). In contrast, a major strength is the Swedish healthcare system, which is public, tax funded, and offers universal access. This significantly reduces the risk of selection bias and ensures a high coverage of cases. The well-characterized cohort of SLE patients followed by a limited number of experienced rheumatologists at a single tertiary referral center constituted another strength of the study. Finally, the SARS-CoV-2 antibodies were not only quantified but their neutralizing capacity was also evaluated.

To conclude, we show that a large proportion of Swedish SLE patients have serological signs of exposure to SARS-CoV-2 prior to vaccination but apparently with a minor impact on the SLE course. SARS-CoV-2 antibodies, particularly IgA and IgM, had low neutralizing capacity and were detected also in samples obtained prior to the pandemic. Except for anti-Sm, specific SLE autoantibodies did not associate with SARS-CoV-2 IgG. The use of steroids and DMARDs showed no distinct effects on the ability to mount an antibody response to SARS-CoV-2 and self-reported COVID-19 symptoms (except for fever) correlated poorly with all detected antibody isotypes.

## Data Availability Statement

The raw data supporting the conclusions of this article will be made available by the authors, without undue reservation.

## Ethics Statement

The study protocol was approved by the regional ethics review board in Linköping (Decision number M75-08/2008). The patients/participants provided their written informed consent to participate in this study.

## Author Contributions

JS: drafting the manuscript and revising it critically for important intellectual content; access to all the data in the study and takes responsibility for the integrity of the data and the accuracy of the data analysis; study conception and design; data validation; acquisition of data, analysis, and interpretation of data; and approval of the final version to be published. MA: drafting the manuscript and revising it critically for important intellectual content; acquisition of data, analysis, and interpretation of data; and approval of the final version to be published. MF: drafting the manuscript and revising it critically for important intellectual content; access to all the data in the study and takes responsibility for the integrity of the data and the accuracy of the data analysis; acquisition of data, analysis, and interpretation of data; and approval of the final version to be published. YZ: drafting the manuscript and revising it critically for important intellectual content; acquisition of data, analysis, and interpretation of data; and approval of the final version to be published. LS: drafting the manuscript and revising it critically for important intellectual content; acquisition of data, analysis, and interpretation of data; and approval of the final version to be published. CD: drafting the manuscript and revising it critically for important intellectual content; study conception and design; data validation; acquisition of data, analysis, and interpretation of data; and approval of the final version to be published. JH: drafting the manuscript and revising it critically for important intellectual content; study conception and design; data validation; acquisition of data, analysis, and interpretation of data; and approval of the final version to be published. CS: writing the original draft; drafting the manuscript and revising it critically for important intellectual content; access to all the data in the study and takes responsibility for the integrity of the data and the accuracy of the data analysis; study conception and design; data validation; acquisition of data, analysis, and interpretation of data; and approval of the final version to be published. All authors contributed to the article and approved the submitted version.

## Funding

This work was supported by grants from MIIC at Linköping University, the Swedish Rheumatism Association, the Region Östergötland (ALF Grants), the Swedish Society of Medicine, the Gustafsson Foundation, the King Gustaf V’s 80-year Anniversary Foundation and the King Gustaf V and Queen Victoria’s Freemasons Foundation.

## Conflict of Interest

The authors declare that the research was conducted in the absence of any commercial or financial relationships that could be construed as a potential conflict of interest.

## Publisher’s Note

All claims expressed in this article are solely those of the authors and do not necessarily represent those of their affiliated organizations, or those of the publisher, the editors and the reviewers. Any product that may be evaluated in this article, or claim that may be made by its manufacturer, is not guaranteed or endorsed by the publisher.
