# Supplementary material for: SARS-CoV-2 Antibody Isotypes in Systemic Lupus Erythematosus Patients Prior to Vaccination: Associations With Disease Activity, Antinuclear Antibodies, and Immunomodulatory Drugs During the First Year of the Pandemic
Source: Front Immunol. 2021 Aug 27;12:724047. doi: 10.3389/fimmu.2021.724047 (PMC8430325; doi:10.3389/fimmu.2021.724047)
Supplement: Supplementary file 1 [file Presentation_1.pptx]

## Slide 1
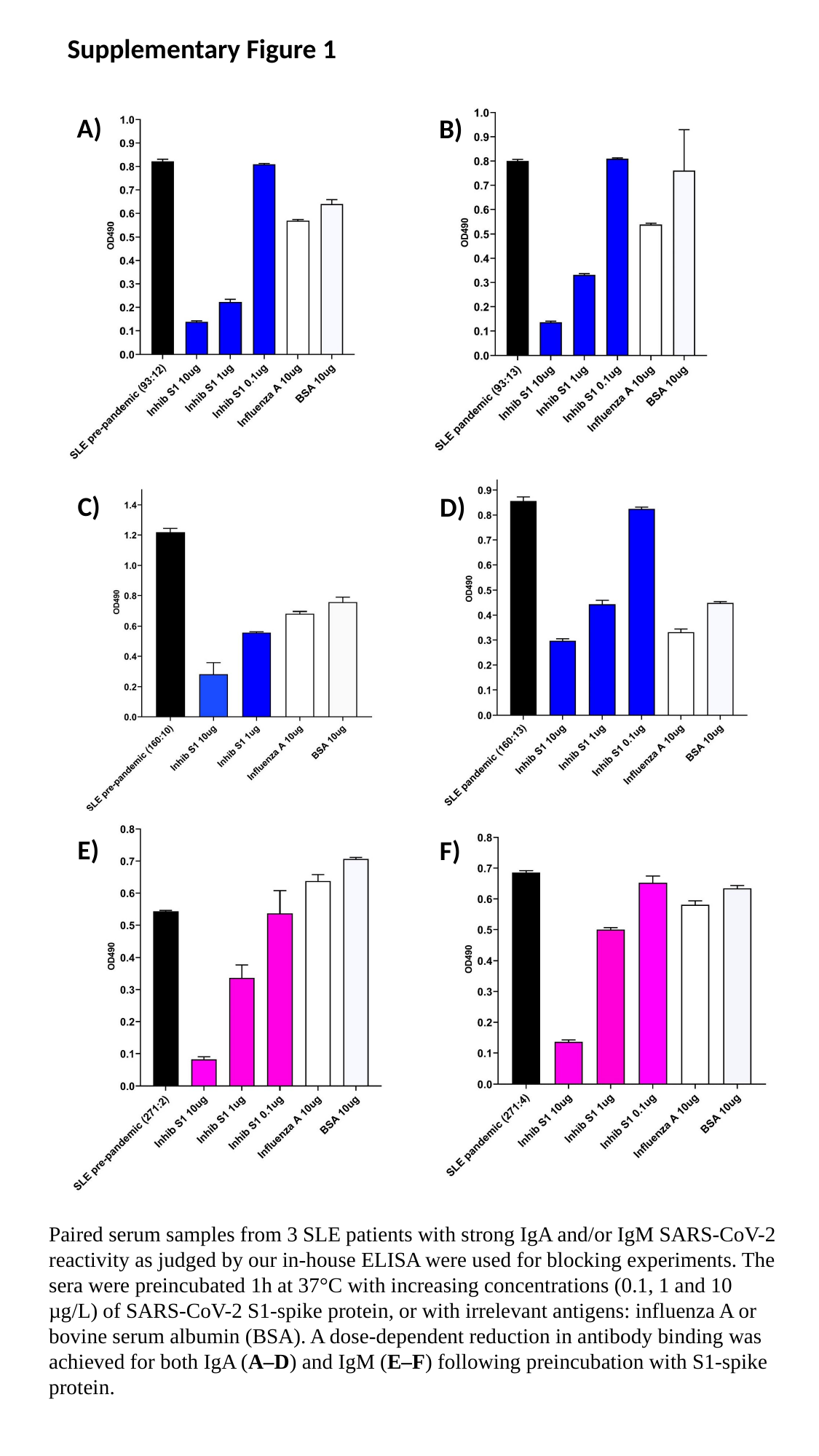

Supplementary Figure 1
A)
B)
C)
D)
E)
F)
E)
F)
Paired serum samples from 3 SLE patients with strong IgA and/or IgM SARS-CoV-2 reactivity as judged by our in-house ELISA were used for blocking experiments. The sera were preincubated 1h at 37°C with increasing concentrations (0.1, 1 and 10 µg/L) of SARS-CoV-2 S1-spike protein, or with irrelevant antigens: influenza A or bovine serum albumin (BSA). A dose-dependent reduction in antibody binding was achieved for both IgA (A–D) and IgM (E–F) following preincubation with S1-spike protein.
