# Supplementary material for: SARS-CoV-2 Antibody Isotypes in Systemic Lupus Erythematosus Patients Prior to Vaccination: Associations With Disease Activity, Antinuclear Antibodies, and Immunomodulatory Drugs During the First Year of the Pandemic
Source: Front Immunol. 2021 Aug 27;12:724047. doi: 10.3389/fimmu.2021.724047 (PMC8430325; doi:10.3389/fimmu.2021.724047)
Supplement: Supplementary file 2 [file Presentation_2.pptx]

## Slide 1
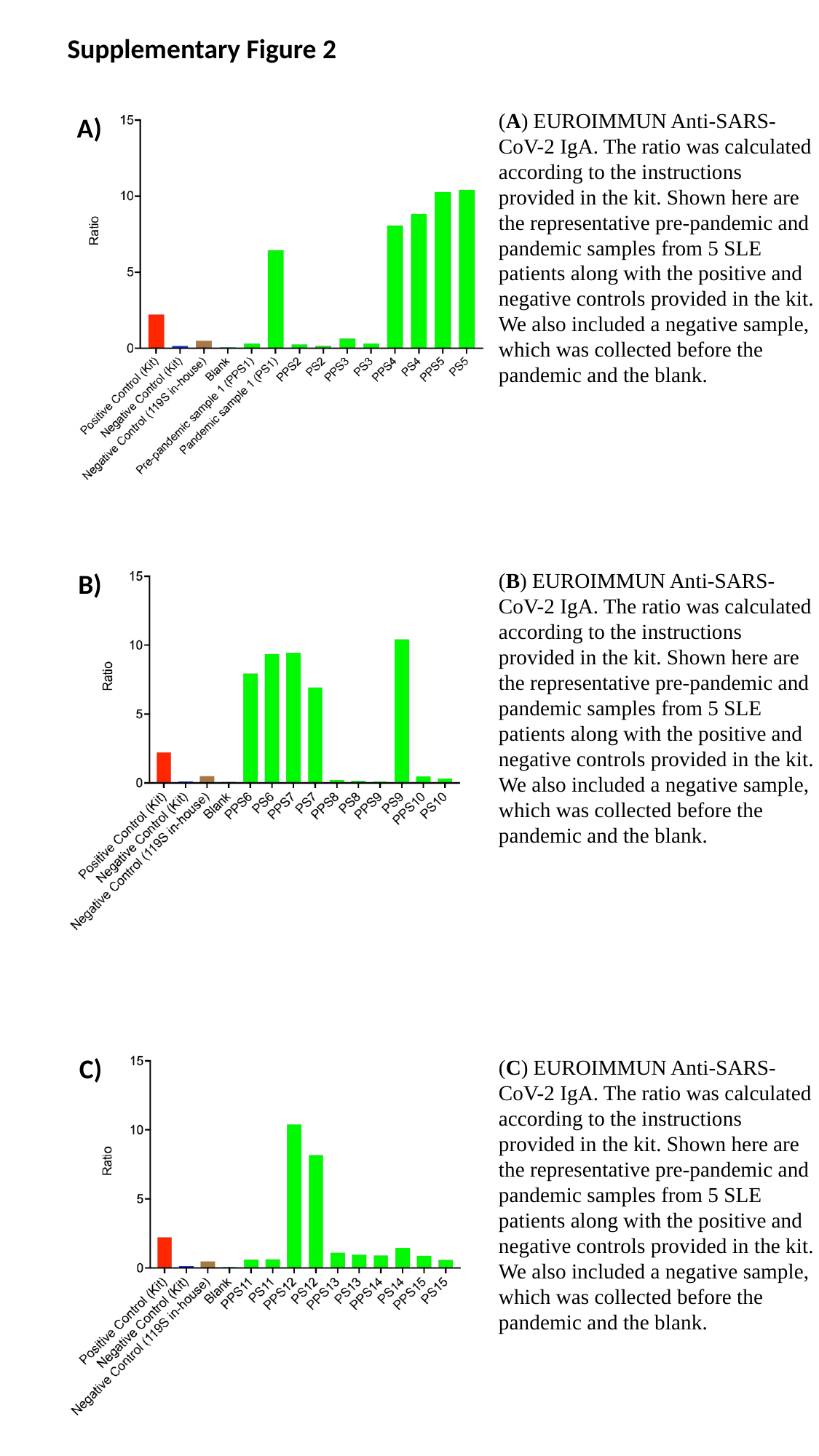

Supplementary Figure 2
(A) EUROIMMUN Anti-SARS-CoV-2 IgA. The ratio was calculated according to the instructions provided in the kit. Shown here are the representative pre-pandemic and pandemic samples from 5 SLE patients along with the positive and negative controls provided in the kit. We also included a negative sample, which was collected before the pandemic and the blank.
A)
B)
(B) EUROIMMUN Anti-SARS-CoV-2 IgA. The ratio was calculated according to the instructions provided in the kit. Shown here are the representative pre-pandemic and pandemic samples from 5 SLE patients along with the positive and negative controls provided in the kit. We also included a negative sample, which was collected before the pandemic and the blank.
C)
(C) EUROIMMUN Anti-SARS-CoV-2 IgA. The ratio was calculated according to the instructions provided in the kit. Shown here are the representative pre-pandemic and pandemic samples from 5 SLE patients along with the positive and negative controls provided in the kit. We also included a negative sample, which was collected before the pandemic and the blank.

## Slide 2
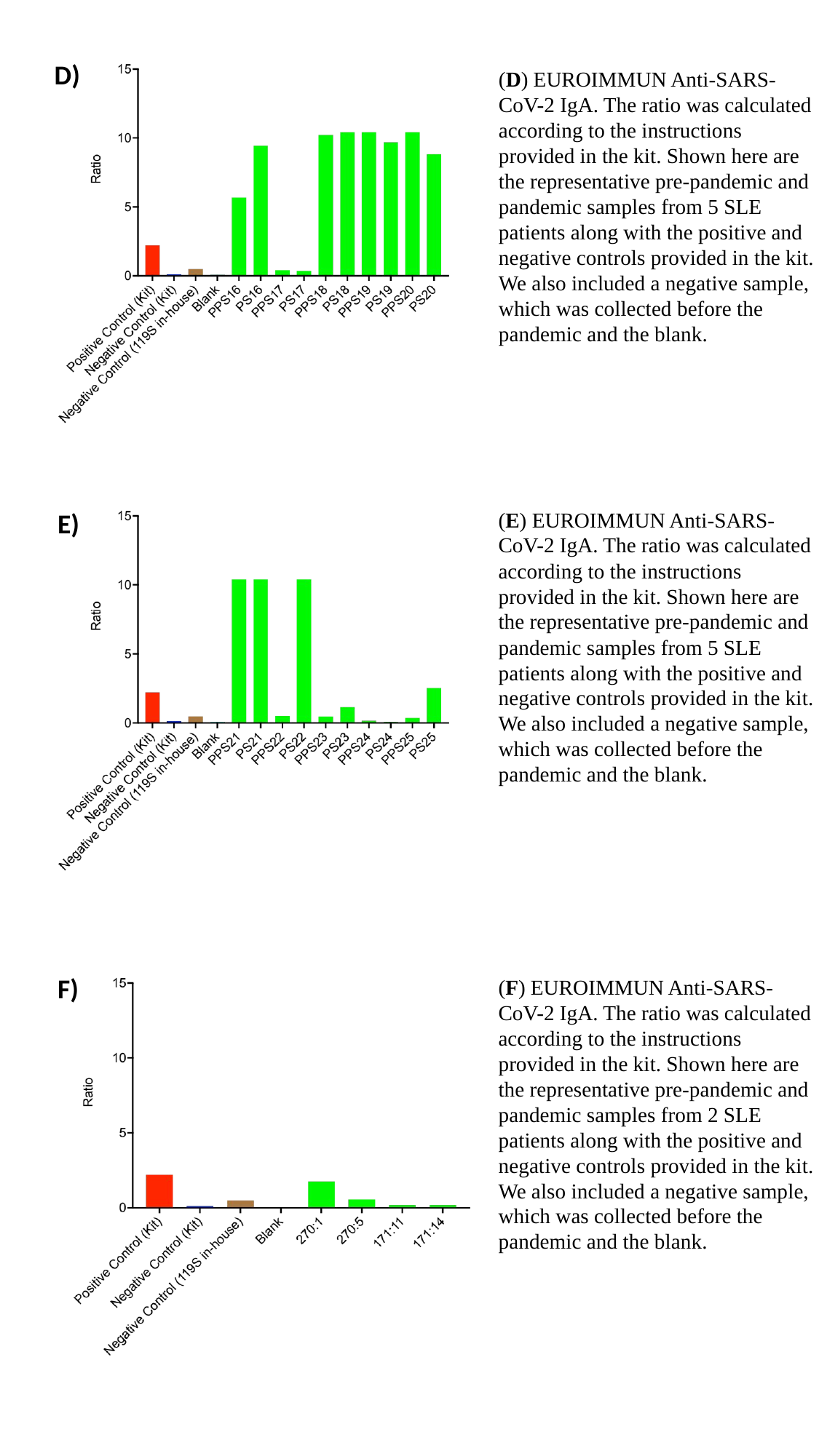

D)
(D) EUROIMMUN Anti-SARS-CoV-2 IgA. The ratio was calculated according to the instructions provided in the kit. Shown here are the representative pre-pandemic and pandemic samples from 5 SLE patients along with the positive and negative controls provided in the kit. We also included a negative sample, which was collected before the pandemic and the blank.
E)
(E) EUROIMMUN Anti-SARS-CoV-2 IgA. The ratio was calculated according to the instructions provided in the kit. Shown here are the representative pre-pandemic and pandemic samples from 5 SLE patients along with the positive and negative controls provided in the kit. We also included a negative sample, which was collected before the pandemic and the blank.
F)
(F) EUROIMMUN Anti-SARS-CoV-2 IgA. The ratio was calculated according to the instructions provided in the kit. Shown here are the representative pre-pandemic and pandemic samples from 2 SLE patients along with the positive and negative controls provided in the kit. We also included a negative sample, which was collected before the pandemic and the blank.

## Slide 3
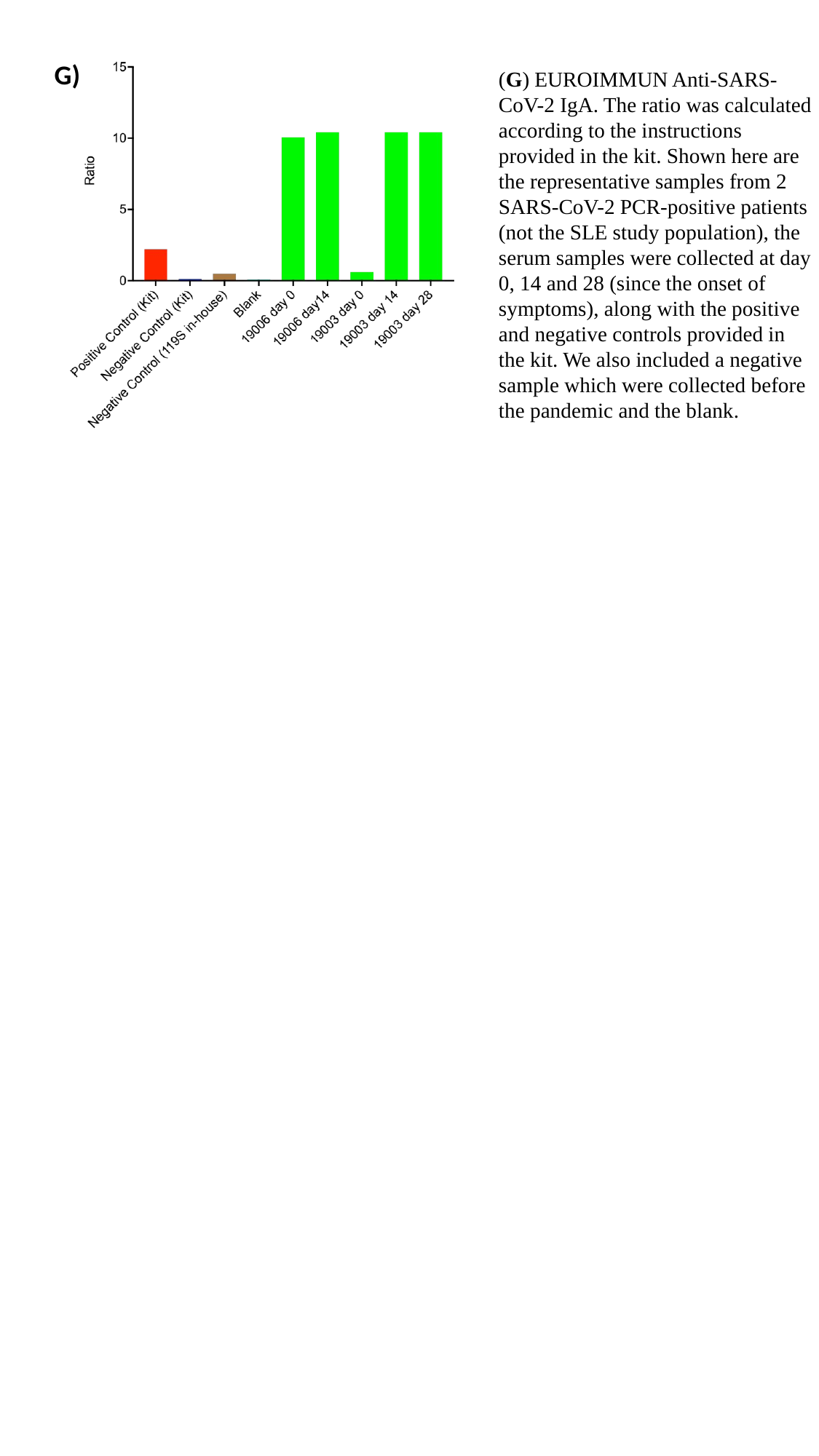

G)
(G) EUROIMMUN Anti-SARS-CoV-2 IgA. The ratio was calculated according to the instructions provided in the kit. Shown here are the representative samples from 2 SARS-CoV-2 PCR-positive patients (not the SLE study population), the serum samples were collected at day 0, 14 and 28 (since the onset of symptoms), along with the positive and negative controls provided in the kit. We also included a negative sample which were collected before the pandemic and the blank.
